# High yield synthesis of graphene quantum dots from biomass waste as a highly selective probe for Fe^3+^ sensing

**DOI:** 10.1038/s41598-020-78070-2

**Published:** 2020-12-04

**Authors:** Aumber Abbas, Tanveer A. Tabish, Steve J. Bull, Tuti Mariana Lim, Anh N. Phan

**Affiliations:** 1 School of Engineering, Newcastle University, Newcastle upon Tyne, NE1 7RU UK; 2 UCL Cancer Institute, University College London, London, WC1E 6DD UK; 3 School of Civil and Environmental Engineering, Nanyang Technological University, Singapore, 639798 Singapore

**Keywords:** Materials science, Nanoscience and technology

## Abstract

Graphene quantum dots (GQDs), a novel type of zero-dimensional fluorescent materials, have gained considerable attention owing to their unique optical properties, size and quantum confinement. However, their high cost and low yield remain open challenges for practical applications. In this work, a low cost, green and renewable biomass resource is utilised for the high yield synthesis of GQDs via microwave treatment. The synthesis approach involves oxidative cutting of short range ordered carbon derived from pyrolysis of biomass waste. The GQDs are successfully synthesised with a high yield of over 84%, the highest value reported to date for biomass derived GQDs. As prepared GQDs are highly hydrophilic and exhibit unique excitation independent photoluminescence emission, attributed to their single-emission fluorescence centre. As prepared GQDs are further modified by simple hydrothermal treatment and exhibit pronounced optical properties with a high quantum yield of 0.23. These modified GQDs are used for the highly selective and sensitive sensing of ferric ions (Fe^3+^). A sensitive sensor is prepared for the selective detection of Fe^3+^ ions with a detection limit of as low as 2.5 × 10^–6^ M. The utilisation of renewable resource along with facile microwave treatment paves the way to sustainable, high yield and cost-effective synthesis of GQDs for practical applications.

## Introduction

Graphene quantum dots (GQDs), as a zero-dimensional derivates of graphene, have ignited tremendous research interests in recent years^1–6^. Due to the prominent quantum confinement^6^ and edge effects^7^, GQDs display excellent optical and electrical properties. GQDs offer significant advantages of low cost, high water solubility, stable fluorescence, tunable bandgap, low toxicity and good biocompatibility^8–11^, and thus making them a legitimate competitor to the traditional semiconductor quantum dots (QDs) (e.g. ZnS, TiO, CdSe, CdS, CdTe), which are expensive, cytotoxic, and show low-biocompatibility^12^. Owing to their unique properties, GQDs exhibit substantial potential for a wide range of promising applications in catalysis, sensors, bio-imaging, medical diagnosis, optoelectronics, and energy storage devices^10,11,13–15^. Therefore, an easy, cost-effective and high yield synthesis of GQDs remains an unmet need for their real-world application.

To date, several carbon-based resources have been used to synthesise GQDs, such as graphite flakes^16^, carbon nanotubes^17^, graphene^18^, carbon fibre^19^, coal^20^ and others^21,22^. A number of synthesis techniques have been adopted such as hydrothermal^18,23^, electrochemical^24,25^, sonochemical^26^, solvothermal^11,27,28^, laser ablation^29^, microwave cutting^30^, etc. Among these approaches, microwave treatment is indeed beneficial due to its uniform and rapid heating, thereby leading to extremely short reaction time^31^. The utilisation of microwave treatment has been reported for the rapid synthesis of GQDs^30,32^. However, most of these reports either used expensive precursor materials, complex processes, highly toxic chemicals or gave very low product yield. For example, Li et al. synthesised greenish-yellow GQDs from graphene oxide to generate a product yield of 8%^30^. Wang et al. reported the synthesis of white light emitting GQDs by two step microwave assisted hydrothermal approach that involved large amount of corrosive acids and required 14 h reaction time^32^. Shin et al. reported the synthesis of GQDs with relatively good yield, but the process involved large amount of corrosive and toxic chemicals (H_2_SO_4_, KMnO_4_)^33^. In another report, Nair et al. synthesised GQDs by the microwave treatment of graphene oxide using strong oxidizing agent (KMnO_4_)^34^. Thus, an easy, rapid, convenient, cost effective, and environmentally friendly approach for the high yield synthesis of GQDs from low cost precursors is highly demanded.

Biomass waste, which is green, cheap, abundant, easily available and rich in carbon, can be considered as a potential precursor for the synthesis of GQDs. Moreover, the disposal and recycling of biomass waste is becoming a major challenge of the modern world. Thus, the use of biomass waste would solve the recycling problem as well as fulfil the need for a cost-effective precursor for the synthesis of GQDs. Herein, biomass waste is proposed as a cheap and sustainable precursor for the significant high-yield production of GQDs for the first time, through a simple microwave approach. Interestingly, the hydrothermally modified form of these GQDs exhibit pronounced optical properties and are utilised in a selective and sensitive detection of ferric ions (Fe^3+^).

The design and development of efficient and selective fluorescent sensors for metal ion detection is highly desirable due to their direct relation with the environment and water pollution^35,36^. Recently, GQDs are emerging as a novel type of fluorescence sensors due to their intriguing properties such as high solubility, low toxicity, chemical inertness, stable photoluminescence (PL), superior surface grafting, and so on^37^. In the context of strong luminescence properties, GQDs have been used to detect different analytes^38–40^. A number of sensors have been developed based on the PL quenching mechanism of GQDs^41–43^. Wang et al. was the first to report on Fe^3+^ ions detection by selective fluorescence quenching of GQDs^43^. Fe^3+^ is a very important metal ion present in both environmental and biological systems. Fe^3+^ ions can coordinate with different type of proteins present in biological systems. The concentration of Fe^3+^ ions plays an important role in the manipulation of Parkinson’s disease (PD) since the accumulation of iron has been found at the affected neurons of PD patients^44^. Moreover, Fe^3+^ ions are among the substantial pollutants present in the environment and inducing water pollution^45^. In response to these reasons, the sensitive and selective detection of Fe^3+^ ions is urgently needed. Instrument-based detection and electrochemical sensing usually require complex preparations and are limited by their reproducibility and reliability^45–47^. Thus, the simple and sensitive detection by a luminescence-based approach make GQDs a very powerful tool for monitoring harmful metal ions in the environment.

In this study, GQDs have been prepared by a simple, yet efficient microwave treatment of carbon rich biochar obtained through the pyrolysis of biomass waste. The GQDs were synthesised in as short as a 30 min time span and exhibit excellent optical properties. A high product yield of ~ 84% was achieved with increasing the reaction time or microwave power. Moreover, these GQDs were modified by simple hydrothermal treatment and displayed enhanced optical properties for the selective and sensitive detection of metal ions. In view of the PL properties of GQDs, a luminescence sensor was designed for Fe^3+^ sensing. It was shown that Fe^3+^ can be detected by GQDs leading to their fluorescence quenching. For the first time, this paper reports Fe^3+^ ion sensing using GQDs synthesised via microwave treatment of spent tea. Under the premise of employing a renewable cost-effective precursor, simple approach, acquiring high yield of GQDs and implementing in practical applications, our study lends further credence to the industrial potential of GQDs. Moreover, this procedure is generic and applicable to any other biomass waste which can yield carbon rich precursor by pyrolysis treatment.

## Results and discussion

### Synthesis and characterisation of GQDs

**Figure 1:**
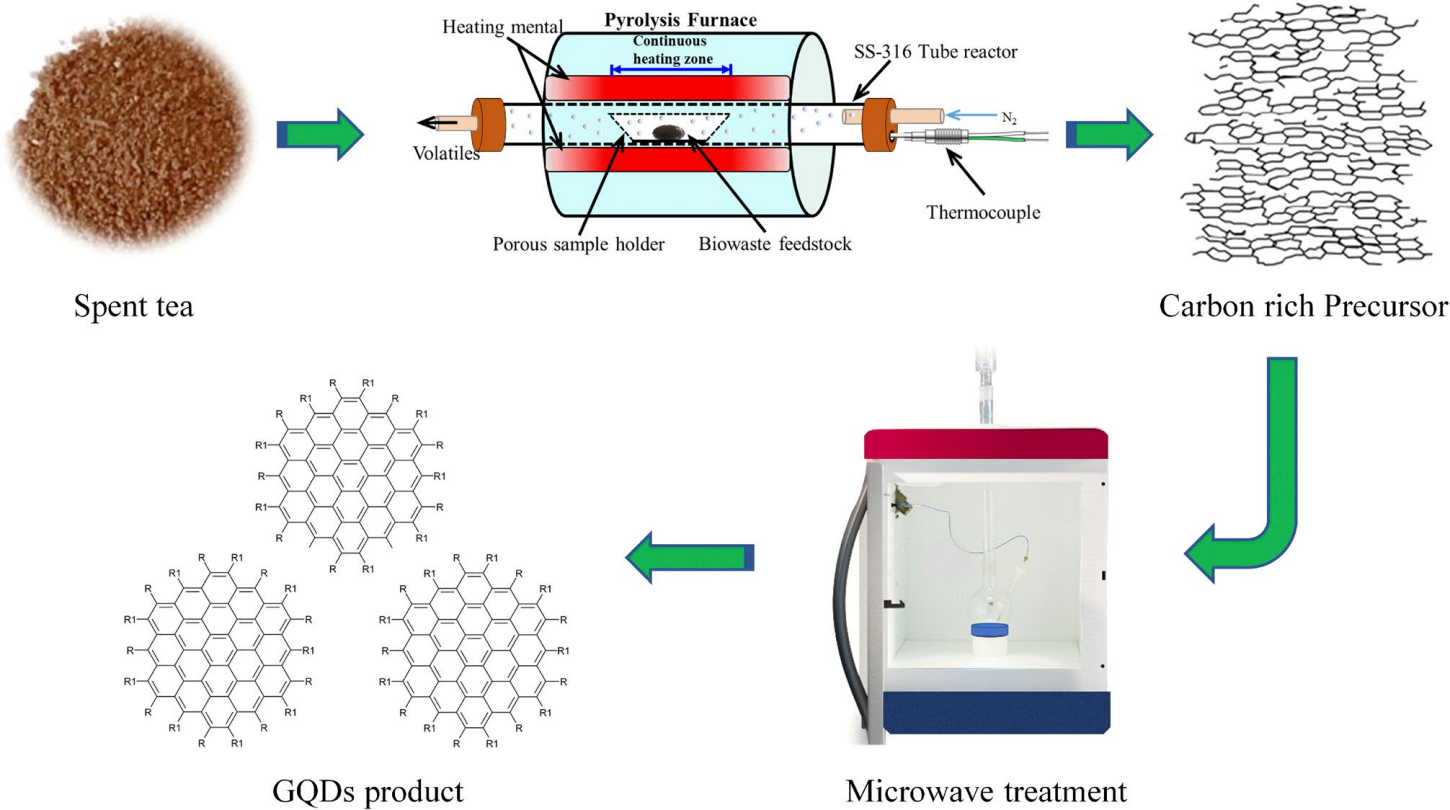
A schematic illustration of the synthesis procedure for graphene quantum dots (GQDs). Spent tea waste is subjected to pyrolysis treatment and carbon rich biochar is obtained, which is then microwave treated to produce GQDs with high yield. Figure was drawn using ChemDraw v16 (https://www.perkinelmer.com), Adobe Photoshop 2019 (https://www.adobe.com) and Microsoft PowerPoint 2016 (https://www.microsoft.com).

GQDs were successfully synthesised by oxidative cutting of a biomass-derived carbon rich precursor using nitric acid (HNO_3_) as an oxidising agent via microwave heating of 15–180 min duration. Figure 1 shows a schematic illustration of the synthesis procedure for GQDs. The products obtained after each microwave treatment were examined by studying their optical properties.

**Figure 2:**
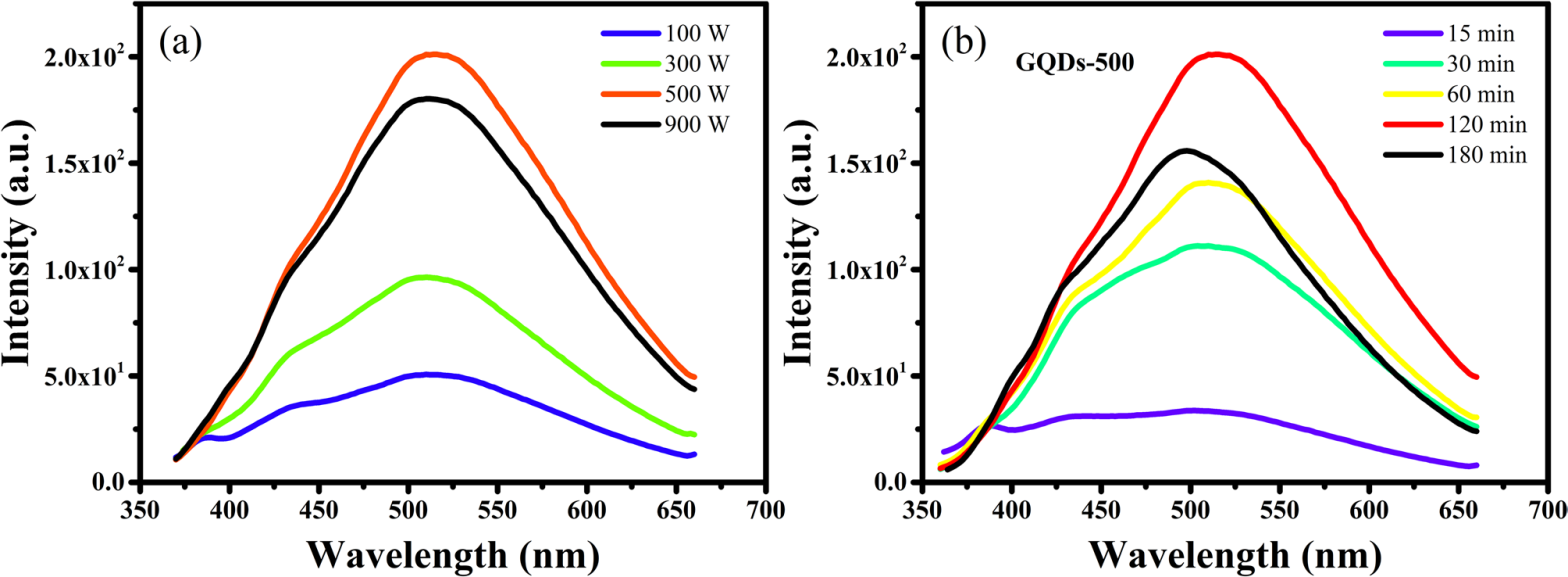
Comparison of the effect of (**a**) microwave power at a fixed duration of 120 min and (**b**) processing time at a fixed power of 500 W, on the photoluminescence (PL) emission of GQDs at an excitation wavelength of 340 nm. Figures were drawn using OriginPro 2018b (https://www.originlab.com).

In order to find out the best operating parameters a thorough study was carried out at a range of microwave powers (100–900 W) and processing times (15–180 min). The optical properties of the products were studied by recording their PL emission. Identical concentrations of 50 μg mL^−1^ were used for comparing the PL intensities of the GQDs prepared at different powers. The PL emission intensities of the products prepared at different microwave powers (100, 300, 500 and 900 W power) for a fixed duration of 120 min, and at a range of treatment times (15, 30, 60, 120 and 180 min) at a fixed power of 500 W, are compared in Fig. 2. The products obtained at 100 W power do not show significant optical emission, indicating that such a low microwave power could not induce cutting of carbon domains to generate GQDs. Therefore, the microwave power was further increased. It can be seen in Fig. 2a that the PL emission intensity increases with power up to 500 W, while it declines with further increase in power to 900 W. Similarly, the PL intensity increases with an increase in the treatment time up to 120 min and decreases thereafter (Fig. 2b). These results suggest that increase in power and process time intensify the cleavage of carbon domains into nanosized GQDs leading to enhanced optical emission, while increasing the power and time over a certain limit may cause the destruction of the surface and structure of GQDs resulting in diminished PL emission. These findings suggest that the intermediate values of 500 W power and 120 min process duration are the best conditions for the synthesis of high quality GQDs with excellent optical properties.

**Figure 3:**
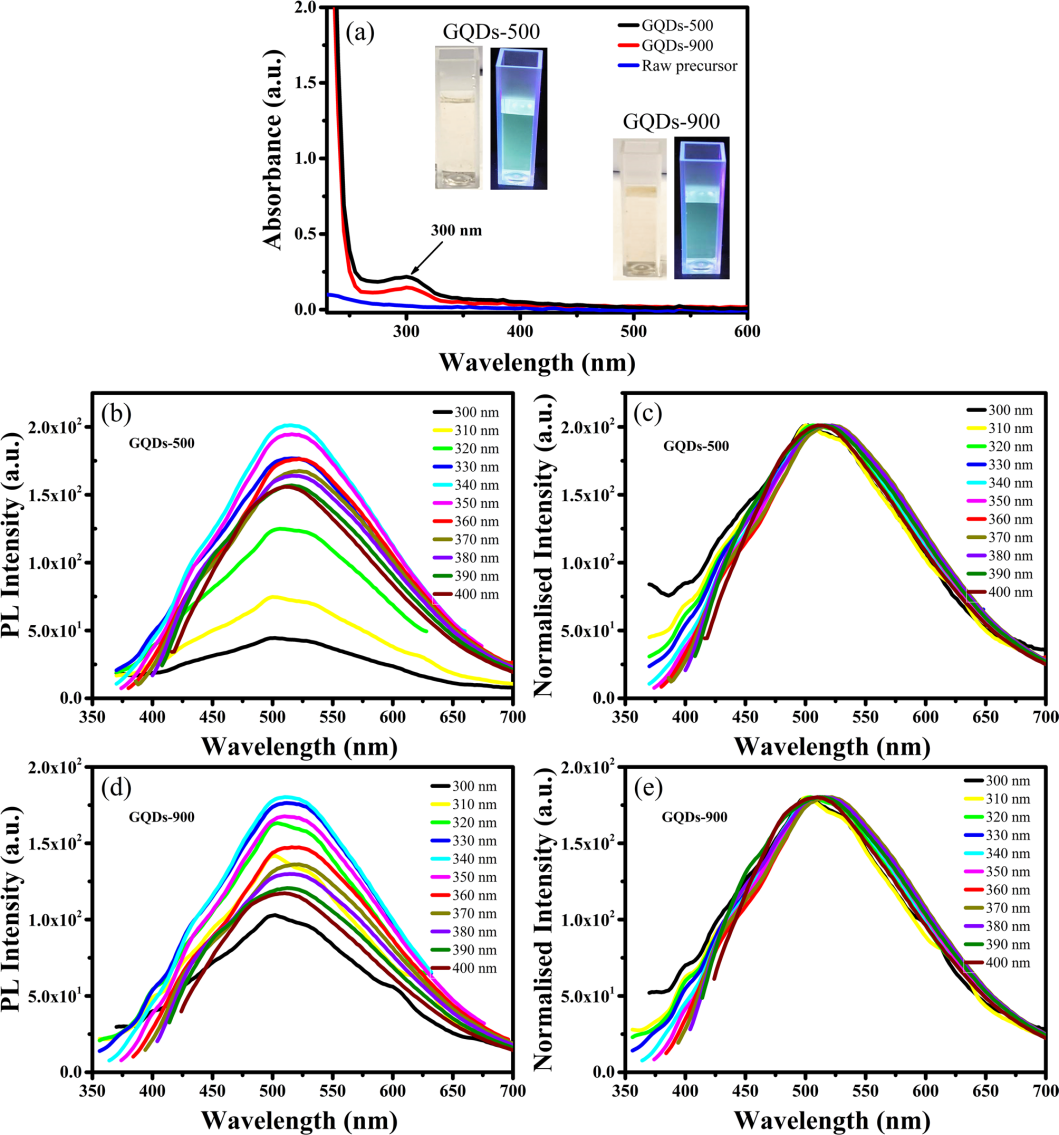
(**a**) Ultraviolet visible (UV–Vis) spectra of raw precursor, GQDs-500 and GQDs-900 showing a strong absorption of GQDs in the UV range (insets are the photographs of GQDs-500 and GQDs-900 under visible and 365 nm UV light). (**b**) PL spectra of GQDs-500 at different excitation wavelengths and (**c**) their normalised PL spectra exhibiting excitation independent emission. (**d**) PL spectra of GQDs-900 and (**e**) their normalised PL spectra showing no shift in PL peak position. Inset images in (**a**) were taken using iPhone Xs Max (https://www.apple.com) and graphics in (**a**)–(**e**) were plotted using OriginPro 2018b (https://www.originlab.com).

For further understanding, the optical properties of GQDs prepared at 500 and 900 W after 120 min of treatment (named as GQDs-500 and GQDs-900) were studied in detail by ultraviolet–visible (UV–Vis) absorption and PL emission spectroscopies. The UV–Vis absorption spectra of raw precursor, GQDs-500 and GQDs-900 are shown in Fig. 3a. The raw materials do not display notable UV absorbance, while both types of GQDs exhibit strong absorption in the UV range. The nature of both GQDs is similar and show absorption at ~ 300 nm which indicates their identical absorbance states. The peaks observed at about 300 nm are due to n → π* transition of the carbonyl group (C=O), generally observed in graphene systems^48^. The non-bonding or ‘n’ electrons are the unpaired electrons on the oxygen of C=O. Therefore, n to π* transition takes place when one of the unpaired oxygen electrons is excited to the antibonding π* orbital^49^. These results suggest that the UV absorbance in these GQDs is related to their surface oxygenated (C=O) states introduced during oxidative cutting with HNO_3_.

Since the absorbance in GQDs-500 and GQDs-900 is related to their identical absorbance states, a minor change in the intensity can be attributed to the difference in concentration of the absorbance states. Hence, the stronger UV absorbance of GQDs-500 can be ascribed to their higher content of C=O groups. A relatively week absorbance in GQDs-900 may be related to the destruction of the surface structure of GQDs-900 at high microwave power because the high temperature treatment removes the oxygen containing functional groups^50^.

The optical bandgap of these GQDs was calculated based on the UV–Vis absorbance spectra using a Tauc plot^51^. The curve was plotted for (α*h*ʋ)^1/γ^ versus photon energy *h*ʋ converted from the UV–Vis spectra, where, α is optical absorption coefficient, h is Planck’s constant, ʋ is frequency of light, A is a constant and E_g_ is optical bandgap. The γ factor depends on the nature of material and is equal to 1/2 for direct bandgap materials and 2 for indirect bandgap materials^52^. A good linear fit was obtained while using γ = ½ and no good fit was obtained when γ = 2 was used, as shown in Fig. S1 (Supplementary Information). These results indicate a direct bandgap of GQDs studied here. The direct bandgap of both types of GQDs was estimated to be ~ 5.12 eV (see Supplementary Information for details).

Insets in Fig. 3a show photographs of the GQDs-500 and GQDs-900 solutions under visible light and 365 nm UV light. Both types of GQDs display a light-yellow colour under visible light while a green fluorescence is observed under UV light. The optical emission of GQDs was explored by carrying out a detailed study of photoluminescence emission at various excitation wavelengths. Figure 3b–e shows the PL spectra of GQDs-500 and GQDs-900 at various excitation wavelengths. Both types of GQDs exhibit excitation independent PL emission at a range of excitation wavelengths from 300 to 400 nm. When excited by 300 nm excitation wavelength, the GQDs-500 display a weak PL emission with a peak at ~ 514 nm. The PL intensity of GQDs-500 increases gradually with increase in the excitation wavelength and exhibit a strongest emission at 340 nm. The PL intensity decreases thereafter and gradually stabilises at about 390 nm. Therefore, the PL intensities at 390 and 400 nm excitation are almost the same. Similarly, GQDs-900 also show PL emission having a peak at ~ 514 nm when excited by 300 nm light. The PL intensity of GQDs-900 also varies with change in the excitation wavelength and shows the strongest emission at 340 nm excitation. These results suggest that both types of these GQDs are similar in nature.

Most of the carbon-based QDs exhibit excitation dependent PL emission^1,8,13,53–56^. Their PL emission peak positions change with the change in excitation wavelength. However, it is interesting to note that GQDs prepared through the present approach demonstrate an excitation independent emission. Though the PL intensity changes with a change in excitation wavelength for both types of GQDs, however, they exhibit excitation independent PL emission. When the excitation wavelength is changed from 300 to 400 nm, the intensity of PL emission increases to reach a maximum at 340 nm excitation, and decreases thereafter, however the peak position does not shift and remains fixed at ~ 514 nm. In order to check the peak shifting, the PL spectra were normalised to the maximum of the strongest emission (340 nm excitation). The normalised PL spectra in Fig. 3c,e show no shifting in peak positions with change in excitation wavelength and this further confirms the independency of PL emission from the excitation wavelength. This characteristic of the present GQDs is different from other carbon based QDs^18,24,56,57^. Therefore, it requires more in-depth understanding of their formation mechanism.

Several mechanisms have been proposed to explain the origin of PL emission of carbon QDs. The most common ones involve oxygenated functional groups, quantum size, zigzag edge sites, defect effects and recombination of electron–hole pairs^58–62^. Most of the GQDs produced so far exhibit excitation dependent emission which is generally attributed to surface emissive traps and defect states^63^. However, the zigzag edge sites, quantum size and recombination of electron–hole pairs are regarded as single emission fluorescence centres^64,65^ and generate excitation independent emission^66^. It was reported^63,67^ that the PL emission from GQDs at 500–530 nm is mainly attributed to the conjugated carbon skeleton containing zigzag edge sites. Thus, the excitation independent emission of the present GQDs at 514 nm may be attributed to the zigzag sites^66^ and n–π* transition of C=O groups, consistent with their absorbance spectra. Therefore, it is suggested that the present GQDs prepared via microwave irradiation do not have considerable emissive traps or defect states and exhibit PL due to single emission fluorescence centre^64,66^. Since the prepared GQDs displays this unique optical characteristic, this makes them ideal for numerous applications such as lasing, display technologies, multi-target bioimaging and encryption^68^.

The effect of microwave power and treatment time on the product yield was systematically studied. The product yield was recorded for each treatment based on the biochar precursor and the results are summarised in Table 1. The product yield of both types of GQDs increases with increasing reaction time.

**Table 1:** Values of the product yield obtained at different microwave powers and time periods.

Microwave power (W)	Microwave duration (min)	Product yield (wt.%)
500	15	5
	30	20
	60	25
	120	73
	180	84.5
900	15	10
	30	33
	60	80
	120	84

The highest product yield of around 84% is achieved for a reaction time of 180 min at microwave power of 500 W or 120 min at 900 W power. These results suggest that higher microwave power could induce rapid cutting of carbon domains into GQDs. To the best of the authors’ knowledge, these are the highest values of product yield reported to date for the synthesis of GQDs from biomass waste. Table 2 summarises the product yield of GQDs derived from various biomass-based precursors studied previously.

**Table 2:** Comparison of the product yield of quantum dots (QDs) derived from various biomass-based precursors.

Precursor	Product	Synthesis approach	Product yield (wt%)	Refs.
Spent tea derived carbon	GQDs	Microwave treatment	5–84.5	Present work
Plant leaf extract	N-GQDs	Hydrothermal treatment	25.2	^69^
Paper lignin	GQDs	Pyrolysis	8.74–18.02	^70^
Neem leaves	GQDs	Pyrolysis	–	^39^
Rice-husk carbon	GQDs	Pyrolysis + Hydrothermal treatment	15	^71^
Coffee grounds	GQDs	Hydrothermal treatment	33	^72^
Durian	S-GQDs	Hydrothermal treatment	6.8%	^23^
Alkali lignin	GQDs	Hydrothermal treatment	21	^73^
Garlic	CQDs	Hydrothermal treatment	–	^74^
Fresh tomato	Carbon dots	Microwave pyrolysis	–	^75^
Coconut shells	CQDs	Hydrothermal treatment	–	^76^
Chitosan	CQDs	Hydrothermal treatment	10	^77^
Taxus leaves	Carbon dots	Hydrothermal treatment	1	^78^

The quantum yields of GQDs were calculated by comparing their integrated PL intensities and absorbance values with that of quinine sulfate. Quinine sulfate was dissolved in 0.1 M H_2_SO_4_ having a quantum yield of 0.54. As-prepared GQDs-500 and GQDs-900 were dissolved in water and concentrations were adjusted to obtain an absorbance value of less than 0.1 in order to avoid re-absorption effects. A 10 mm cuvette was used and slit widths were fixed at 5.0 nm for both excitation and emission. The following equation was used to calculate the quantum yield^79^:

1 $$\upphi =\upphi _{{\text{r}}} \frac{{{\text{I}}\left( {{\text{Ar}}} \right){\text{n}}^{2} }}{{{\text{I}}_{{\text{r}}} \left( {\text{A}} \right){\text{n}}_{{\text{r}}}^{2} }}$$

where, ɸ and ɸ_r_ are the quantum yields of the sample and standard reference, I and I_r_ are the integrated PL intensities of the sample and reference, Ar and A are the absorbance values and n_r_ and n are the refractive indices of the reference and required sample, respectively. The quantum yields of as-prepared GQDs-500 and GQDs-900 obtained after 120 min of treatment were calculated to be 0.09 and 0.07, respectively.

**Figure 4:**
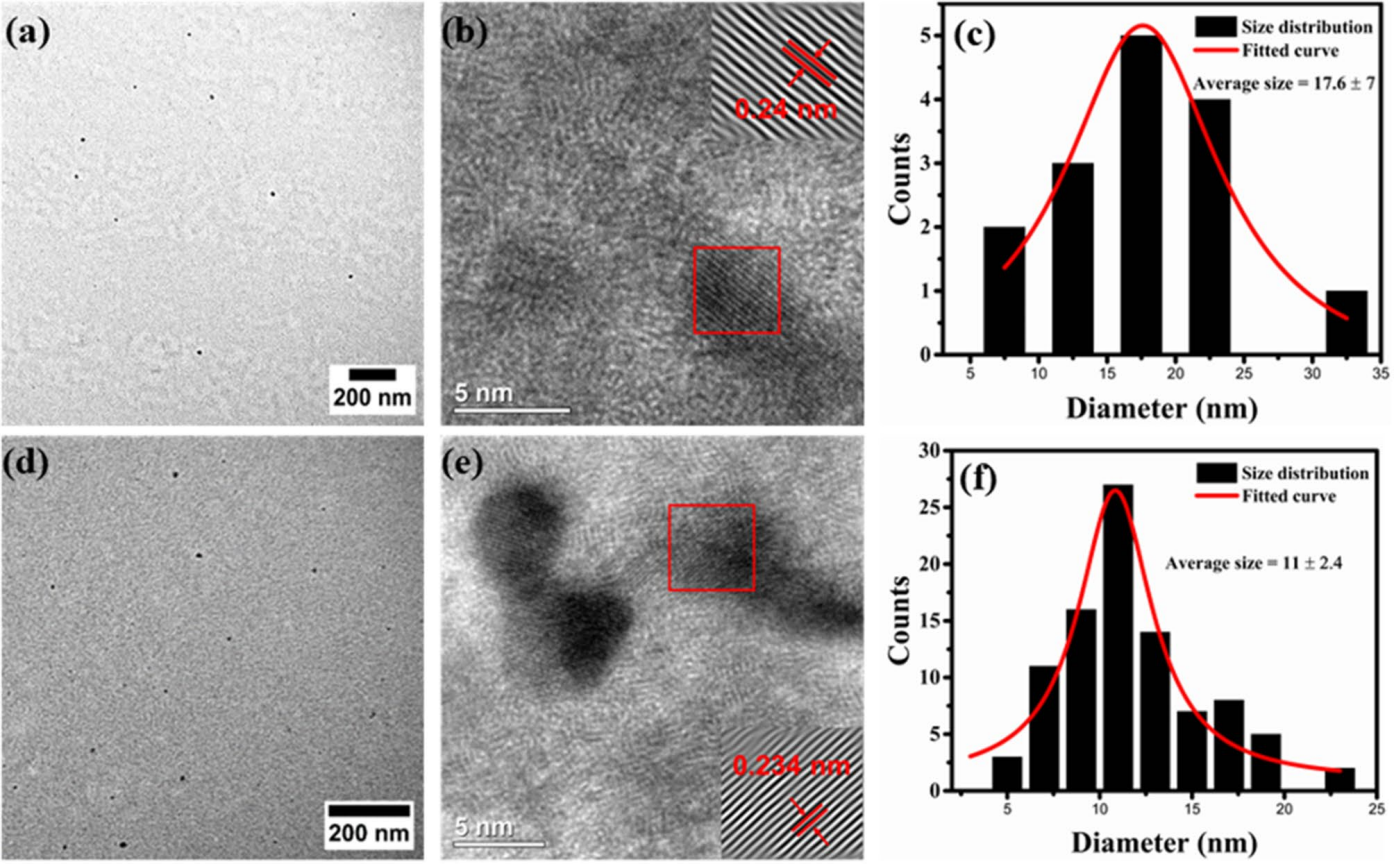
(**a**) TEM image, (**b**) HRTEM image and (**c**) respective particle size distribution of GQDs-500. (**d**) TEM, (**e**) HRTEM and (**f**) size distribution measurements of GQDs-900. Both types of GQDs were obtained after 120 min of treatment. Images in (**a**), (**b**), (**d**) and (**e**) were processed using DigitalMicrograph 3.40.2804.0 (https://www.gatan.com) and plotted using ImageJ 1.8.0_112 (https://imagej.nih.gov/ij/). Figures (**c**) and (**e**) were plotted using OriginPro 2018b (https://www.originlab.com).

Structural studies of GQDs were performed by transmission electron microscopy (TEM). TEM images along with particle size distribution of GQDs-500 and GQDs-900 obtained after 120 min of treatment are presented in Fig. 4. The TEM results verify the synthesis of nanosized GQDs by microwave treatment. The ‘as prepared’ GQDs are well dispersed and possess almost uniform shape. Careful size measurements using the software ‘ImageJ’ show that the GQDs-500 are in the size range of ~ 6 to ~ 34 nm (Fig. 4c). The average size was calculated to be ~ 17.6 nm. The high resolution TEM (HRTEM) image (Fig. 4b) shows the crystalline structure of GQDs and a lattice spacing of ~ 0.24 nm corresponding to (11$\stackrel{-}{2}$0) plane of graphene^71^. An average size below 100 nm, graphene structure and strong optical properties indicate the product obtained is typically GQD^6^. On the other hand, the GQDs-900 exhibit uneven shape with a size range of 4 to 24 nm, with an average size of 11 nm (Fig. 4d–f). The size measurements show that the overall size of GQDs-900 prepared at 900 W is smaller than the size of GQDs-500 prepared at 500 W. This suggests that higher power will break the carbon skeleton more significantly and generate relatively smaller particles. The HRTEM image of GQDs-900 in Fig. 4e displays a lattice spacing of 0.234 nm related to (11$\stackrel{-}{2}$0) plane of graphene (0.24 nm)^71^. These results indicate the graphene structure of both type of GQDs prepared at 500 and 900 W powers. However, higher power had a detrimental effect on the surface and structure of GQDs-900 as noticed in TEM/HRTEM images and variation in the lattice spacing parameter. This structural destruction may partially contribute to a decreased PL emission in GQDs-900. Owing to the superior optical properties and uniform shape and size, GQDs-500 were considered for the further study.

A number of techniques have been implemented to enhance the optical properties and quantum yield of GQDs such as heteroatom doping, surface-functionalisation, size modification, etc^1,3^. Hydrothermal treatment is a simple yet efficient approach for reducing the size of GQDs^18^. Hence, this approach was adopted to modify the size of GQDs-500 and study its effect on the optical properties. This process provides an insight into the enhancement of optical properties of GQDs prepared by different approaches.

### Property and structural studies of modified GQDs

**Figure 5:**
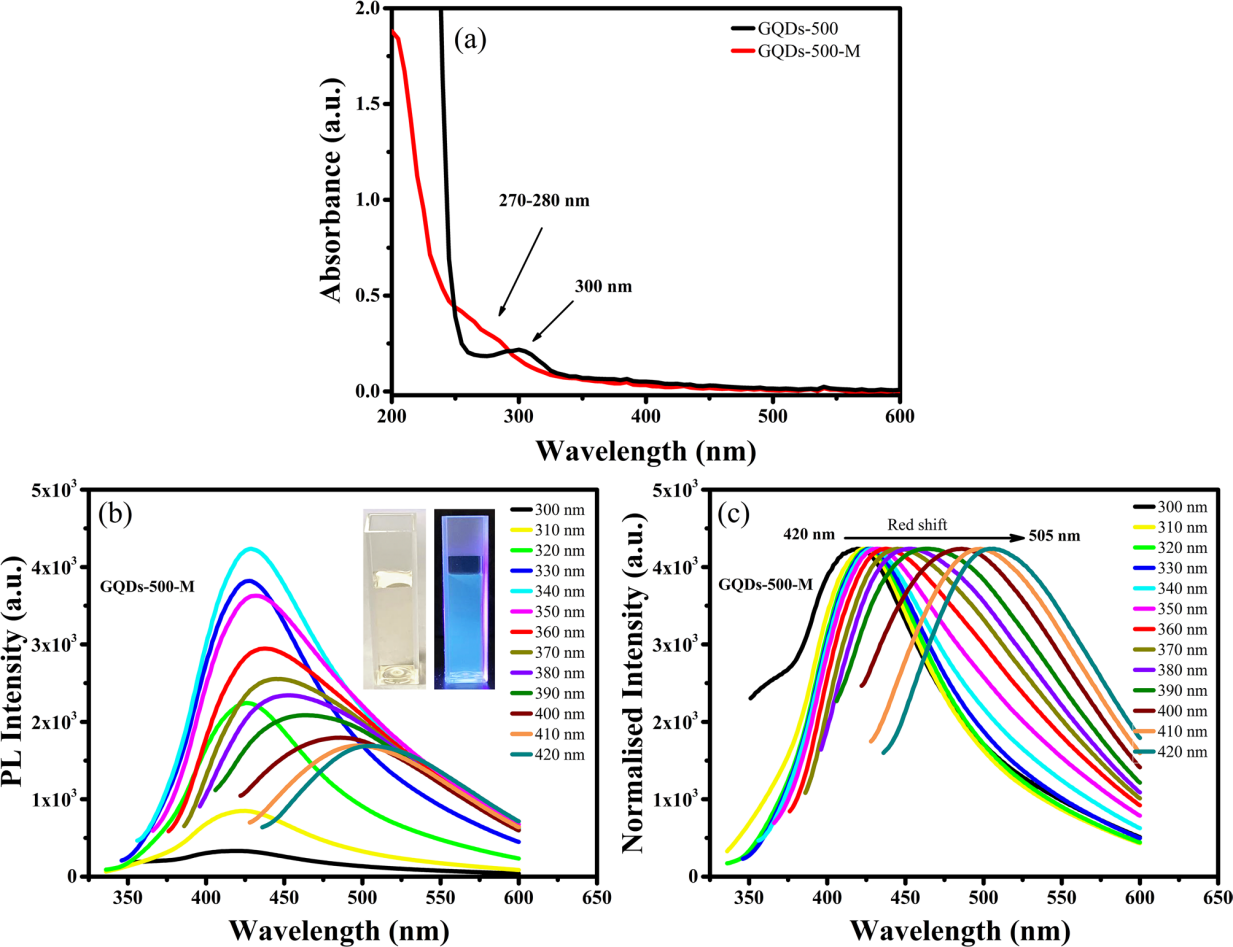
(**a**) UV–Vis spectra of GQDs-500 and GQDs-500-M showing a strong absorption at 300 and 270–280 nm range, respectively. (**b**) The PL spectra of GQDs-500-M at a range of excitation wavelengths (insets are the photographs of GQDs-500-M solution under visible and 365 nm UV light) and (**c**) normalised PL spectra showing a red shift in PL emission. Figures (**a**)–(**c**) were plotted using OriginPro 2018b (https://www.originlab.com) and inset photographs in (**b**) were taken using iPhone Xs Max (https://www.apple.com).

The GQDs-500 were further modified by hydrothermal treatment and denoted as “GQDs-500-M”, where ‘M’ indicates the modified version. The GQDs-500-M were dissolved in water with a concentration of 50 μg mL^−1^ and their optical properties were comprehensively studied by UV–Vis and PL spectroscopies. The UV–Vis spectra of GQDs-500 and GQDs-500-M are displayed in Fig. 5a. The results indicate a strong UV absorption of GQDs-500-M, consistent with the observations of their unmodified forms. The absorption peaks are observed at ~ 270–280 nm, which are attributed to π–π* transition of C=C in aromatics^80^, indicating graphene structure of the GQDs. The optical bandgap of GQDs-500-M was calculated using Tauc plot derived from the absorbance spectrum and determined to be ~ 5.4 eV (see Fig. S1, Supplementary Information). It is worth noting that the bandgap is increased after modification. Moreover, the absorbance spectrum of GQDs-500-M shows a blue shift as compared to their unmodified counterparts. These results indicate a change in optical properties of GQDs after modification.

In order to get further insight into the optical properties, the GQDs-500-M were characterised by PL spectroscopy. The PL emission spectra of GQDs-500-M are presented in Fig. 5b. Inset in Fig. 5b shows photographs of the GQDs-500-M solutions under visible light and 365 nm UV light. The GQDs-500-M solution displays a light-yellow transparent colour under visible light, while a strong blue fluorescence is observed when illuminated by 365 nm UV light. The optical emission of GQDs-500-M was recorded by exposing to a range of excitation wavelengths from 300 to 400 nm and measuring the corresponding emission spectra. When excited by 300 nm laser, the GQDs-500-M show emission at ~ 420 nm. The emission wavelength is higher than the excitation, which is known as Stokes shift. The fluorescence intensity increased with increasing the excitation wavelength up to an optimum level and then diminished. The strongest emission was observed at ~ 430 nm when excited by 340 nm laser. Importantly, the emission peak position changes with the change in excitation wavelength (Fig. 5c). Consequently, the GQDs-500-M exhibit broad emission spectra from a shorter wavelength (blue) of 400 nm to a longer wavelength (near-red) of 550 nm. These results imply the excitation dependent PL emission characteristic of GQDs-500-M. This property suggests the use of these GQDs in multicolour imaging applications (such as bioimaging).

In contrast with unmodified GQDs, the common mechanism suggested for excitation dependent emission in GQDs-500-M is quantum confinement effect, surface emissive traps or different types of functional groups introducing various emissive states which are excited by photons of different energies, resulting in excitation dependent PL emission^4,30,39^. The quantum yield of GQDs-500-M was calculated using Eq. (1) and found to be 0.23. The quantum yield of GQDs-500 was significantly improved after modification, thus highlighting the substantial advantages of improving optical properties using simple hydrothermal treatment.

**Figure 6:**
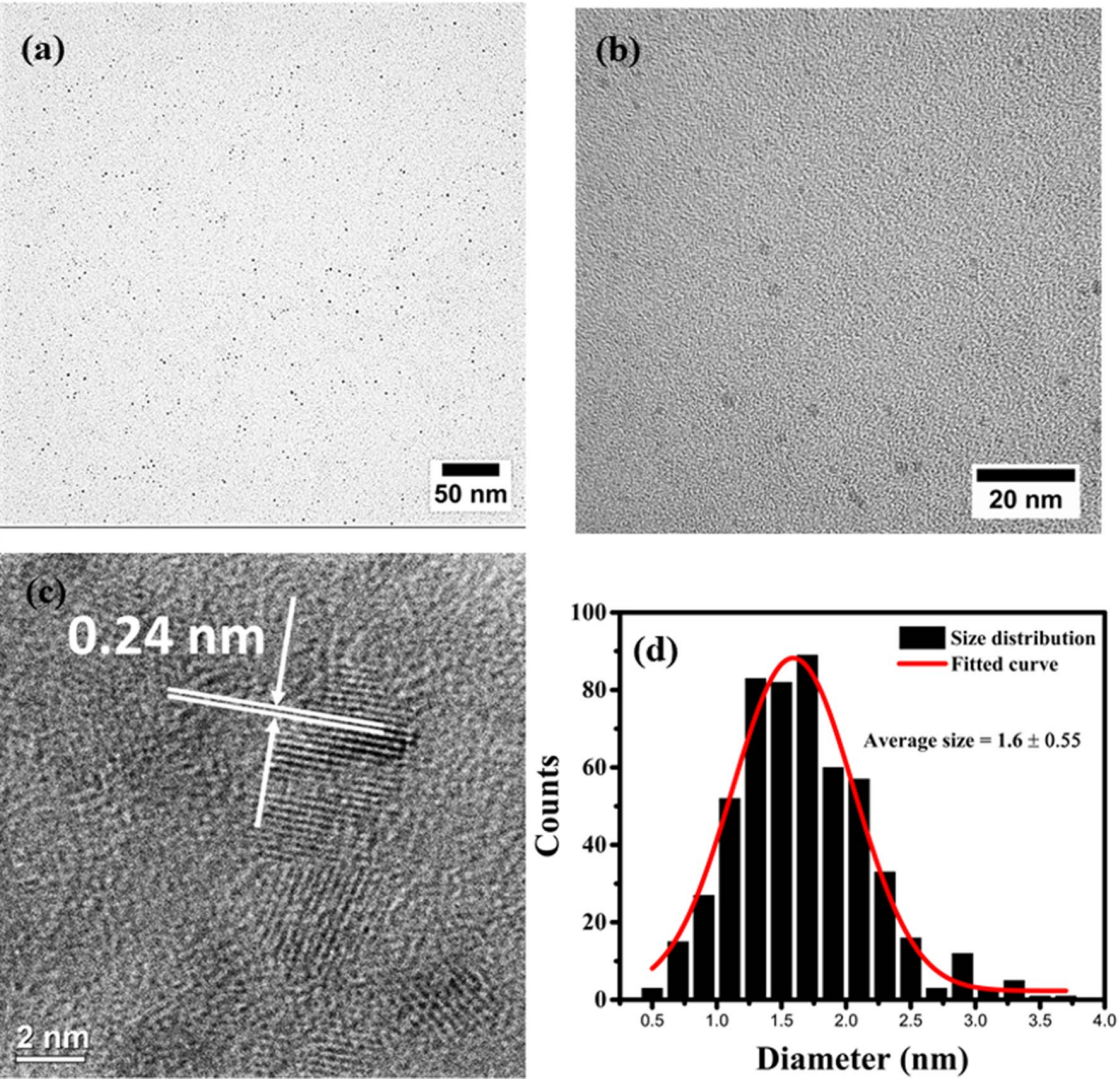
(**a**) Low magnification TEM image, (**b**) high magnification TEM image (**c**) high resolution TEM (HRTEM) image and (**d**) corresponding size distribution of the GQDs-500-M. Images in (**a**), (**b**) and (**c**) were processed using DigitalMicrograph 3.40.2804.0 (https://www.gatan.com) and plotted using ImageJ 1.8.0_112 (https://imagej.nih.gov/ij/). Figure (**d**) was plotted using OriginPro 2018b (https://www.originlab.com).

The structure and size distribution of modified GQDs were studied by TEM analysis. Low and high magnification TEM images of the GQDs-500-M are presented in Fig. 6, which show a uniform dispersion of modified GQDs. The images show a drastic decrease in the size of modified GQDs as compared to their unmodified form (GQDs-500). The high magnification TEM image (Fig. 6b) further shows that the particles are well dispersed and exist in a narrow size range. The size distribution of these GQDs was calculated using ImageJ and the results are presented in Fig. 6d. The results show that the size of GQDs-500-M falls in the range of 0.5 to 3.5 nm, with an average of 1.6 ± 0.55 nm. The results demonstrate a very narrow size distribution. The high resolution TEM image (Fig. 6c) reveals the crystalline structure of modified GQDs. The lattice spacing were calculated to be ~ 0.24 nm corresponding to (11$\stackrel{-}{2}$0) plane of graphene^71^.

**Figure 7:**
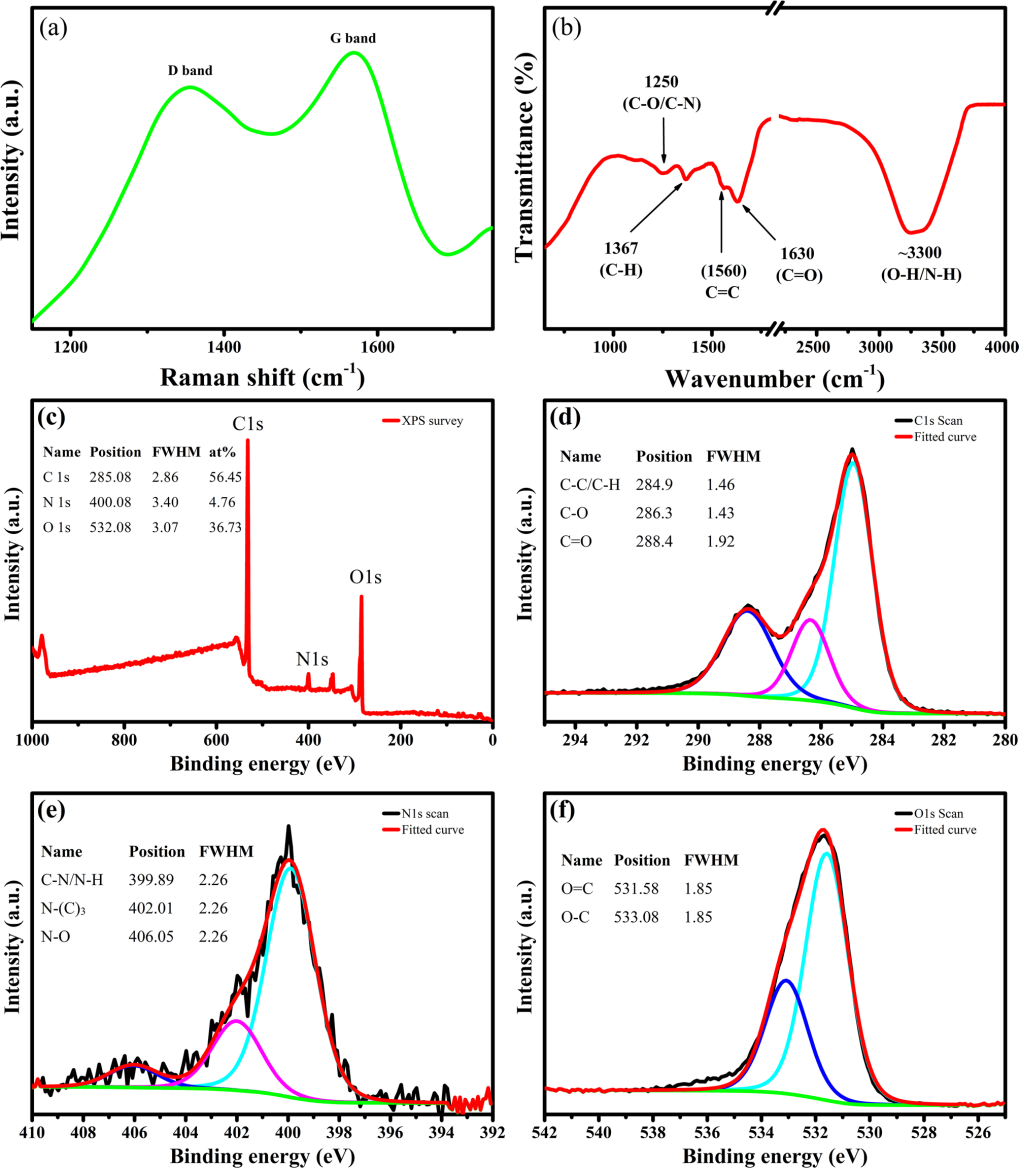
(**a**) Raman and (**b**) FTIR spectra of GQDs-500-M displaying the oxygen and nitrogen functional groups. (**c**) X-ray photoelectron spectroscopy (XPS) survey spectrum, (**d**) C1s, (**e**) N1s and (**f**) O1s high resolution spectra of GQDs-500-M, showing extensive number of oxygen and nitrogen functionalities. Drawings were plotted using OriginPro 2018b (https://www.originlab.com).

The structural characterisation of GQDs was performed by Raman and Fourier transform infrared (FTIR) analysis. The Raman spectrum (Fig. 7) of GQDs-500-M consists of two distinctive bands at about 1356 and 1569 cm^−1^, known as disorder (D) band and crystalline (G) band, respectively. The D and G bands in the range of 1200–1800 cm^−1^ are indicative of a carbonaceous structure^81,82^. The D band is related to the defect mediated zone-edge (near K-point) phonons that shows the defects, edges and disorder in the carbon lattice.

The G band is associated with the in-plane vibration of sp^2^ carbon atoms and usually indicates the crystallinity of the carbon^83^. Moreover, the intensity ratio of D band and G band (I_D_/I_G_) shows the density of disorder in the structure of carbon^83,84^. The I_D_/I_G_ value of GQDs-500-M is ~ 0.98, which show their defective structure, possibly arising from the edge states at the periphery of GQDs^81^. These results are in good agreement with previously reported GQDs^53,54,81^.

Figure 7b shows the FTIR spectrum of GQDs-500-M. The spectrum shows a broad band at ~ 3300 cm^−1^ corresponding to the stretching vibrations of hydroxyl (O–H) and amine (N–H) groups. The peaks at ~ 1630 and ~ 1560 cm^−1^ are related to the stretching vibrations of C=O in carbonyl groups and stretching vibrations of C=C in aromatic groups, respectively. The multiple peaks at 1350–1500 cm^−1^ are attributed to the bending vibrations of C–C/C-H in alkanes. The bands at 1100–1250 cm^−1^ are due to stretching vibrations of C–N groups in amines and C-O groups in ethers, esters, carboxylic acids and alcohols. A broad band at ~ 1000 cm^−1^ corresponds to the C-O stretching of ether groups^85,86^. These results suggest large amounts of oxygen and nitrogen functional groups on the surface of GQDs-500-M. These functional groups make them hydrophilic in nature and render them highly soluble in water.

The surface functionalisation and elemental composition of the GQDs-500-M were further confirmed by X-ray photoelectron spectroscopy (XPS). The survey XPS spectrum shows three major peaks at 285.08, 400.08 and 532.08 eV, which are assigned to C1s, N1s and O1s, respectively (Fig. 7c). Another small peak at 347.08 eV associated with Ca 2p is also observed. The XPS elemental analysis reveals that the GQDs-500-M are mainly composed of C (56.45 at.%), O (36.73 at.%) and N (4.76 at.%), with a limited content of Ca from the feedstock. Ash content in the biomass derived biochar precursor is the origin of calcium and it is present as a CaCO_3_-like species^87^.

The high resolution C1s spectrum (Fig. 7d) can be deconvoluted into three peaks at ~ 284.9, ~ 286.3, and ~ 288.4 eV, corresponding to C–C/C=C, C–O–C and O–C=O groups, respectively^60^. The N1s spectrum (Fig. 7e) can be fitted into three peaks at ~ 399.89, ~ 402.01 and 406.05 eV, which can be attributed to the pyridinic/pyrrolic nitrogen (C–N/N–H), graphitic nitrogen (N-(C)_3_) and nitro (N–O) species, respectively^78,88^. The O1s spectrum (Fig. 7f) consists of two major peaks at 531.58 and 533.08 eV, which can be ascribed to O=C and O–C, respectively^60,89^. These results suggest that the hydrothermal treatment not only modified the size of GQDs but also incorporated extensive content of oxygen and nitrogen functional groups in the matrix of modified GQDs, making them highly soluble in water with good stability. Moreover, no detectable changes were observed in the GQDs-500-M after storing for two months which further verifies their good stability.

### Application as a PL sensor

The as-synthesised GQDs-500-M exhibit strong PL emission and specific oxygenated surface functionalisation. The presence of these functional groups result in the hydrophilicity of the GQDs leading to good solubility in water^55,90,91^. Owing to the presence of these functional groups and strong emission, these GQDs are expected to be an ideal candidate for fluorescence sensing.

Among the various heavy metal ions, the detection and removal of Fe^3+^ ions has always attracted extensive attention^53,92,93^. Generally, iron plays a key role in human life and its deficiency requires considerable attention among the global micronutrient deficiencies. Moreover, the iron percentage has to be monitored and balanced in biological and environmental systems^94^. To achieve this, numerous researchers employed the quenching phenomenon of carbon QDs by the addition of Fe^3+^^4,53,95^. The quenching percentage is related to the concentration of Fe^3+^.

**Figure 8:**
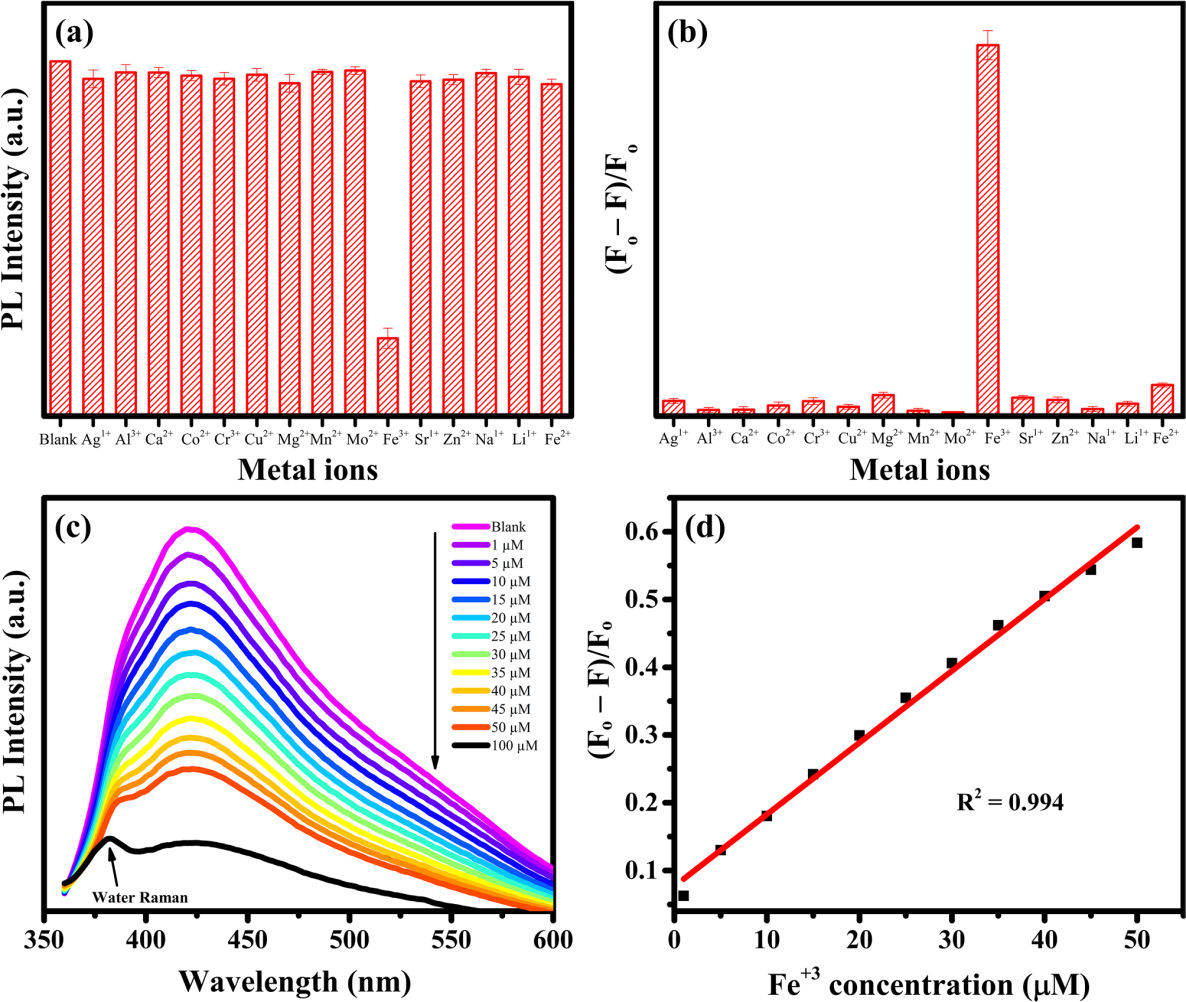
(**a**) Comparison of the PL intensities of 50 μg mL^−1^ GQDs-500-M solution in the presence of different metal ions (100 µM) at an excitation wavelength of 340 nm. (**b**) The comparison of the affinity of different metal ions towards GQDs-500-M (F_0_ and F are the PL intensities of GQDs-500-M without and with 100 µM of different metal ions). (**c**) The PL spectra of GQDs-500-M at different concentrations of Fe^3+^ and (**d**) corresponding linear plot. Three independent experiments were carried out to obtain the mean values. Figures were drawn using OriginPro 2018b (https://www.originlab.com).

In this study, the selective sensing of Fe^3+^ was performed by 50 μg mL^−1^ GQDs-500-M solution using 100 µM concentration of fifteen different metal ions: Ag^1+^, Al^3+^, Ca^2+^, Co^2+^, Cr^3+^, Cu^2+^, Mg^2+^, Mn^2+^, Mo^2+^, Fe^3+^, Sr^1+^, Zn^2+^, Na^1+^, Li^1+^ and Fe^2+^. As discussed earlier, the GQDs-500-M exhibit a strong PL emission when excited by a wavelength of 340 nm. Therefore, this wavelength was selected to monitor the effect of metal ions on the PL intensity. The PL intensities of GQDs-500-M were recorded with the addition of different metal ions. As shown in Fig. 8a, comparing with the control (blank) sample, the PL of GQDs was remarkably quenched only by Fe^3+^ ions among fifteen different metal ions. These results suggest the highly selective detection of Fe^3+^ by the GQDs-500-M.

The mechanism of PL quenching is related to the strong affinity of Fe^3+^ ions towards the hydroxyl/carboxyl groups of GQDs-500-M, leading to a stable complex^13^. PL quenching occurs when the excited electron in GQDs partially transfers to the d orbital of Fe^3+^ instead of undergoing radiative relaxation^13^. The affinity (coordination) of numerous metal ions toward GQDs-500-M was evaluated systematically under similar experimental conditions. Figure 8b shows that the Fe^3+^ ions exhibit strongest affinity towards GQDs-500-M among fifteen different metal ions, suggesting its potential for selective sensing by GQDs-500-M.

Further study was carried out to investigate the effect of Fe^3+^ concentration on the fluorescence intensity of GQDs. The PL spectra of GQDs-500-M with different concentration of Fe^3+^ is shown in Fig. 8c. The spectra indicate that the PL intensity of GQDs-500-M is very sensitive to the concentration of Fe^3+^. The PL intensity decreases with increasing Fe^3+^ concentration. Hence, quenching efficiency of Fe^3+^ was determined by (F_0_ − F)/F_0_, where F_0_ and F are the PL intensities of blank and with different concentrations of metal ions, respectively. A quick PL quenching with Fe^3+^ was observed, therefore the quenching test was performed at low concentration range from 1 to 50 µM of Fe^3+^.

Meanwhile, a good linear plot is observed between the quenching efficiency and Fe^3+^ ions concentration (1–50 µM). As shown in Fig. 8d,a linear regression value (R^2^) of 0.994 was obtained which shows a good fitting and accurate detection of Fe^3+^ ion concentration. These results indicate that the ‘as prepared’ GQDs-500-M can be used as a selective and sensitive sensor for the detection of Fe^3+^ ions. The limit of detection (LOD) and limit of quantification (LOQ) were calculated using the following equations:

2 $$\mathrm{LOD }= 3.3\frac{\upsigma }{\mathrm{S}}$$

3 $$\mathrm{LOQ }= 10\frac{\upsigma }{\mathrm{S}}$$

where σ is the standard deviation of intercept and S is the slop of linear regression plot.

The LOD and LOQ were calculated to be 2.5 ± 0.3 µM and 7.6 ± 0.9 µM, respectively. The detection limit of Fe^3+^ found in this study was much lower than the World Health Organisation (WHO) drinking water guideline limit of Fe^3+^ concentration (5.36 µM)^96^, suggesting that the as prepared GQDs-500-M are promising in detecting trace amounts of Fe^3+^. A comparison of the Fe^3+^ ion detection by different carbon-based materials reported by various researchers is given in Table 3. The detection limit of 2.5 ± 0.3 µM Fe^3+^ observed in this work is considerably lower than the values previously reported for detecting Fe^3+^. Thus, we believe the ‘as prepared’ GQDs-500-M are very promising for Fe^3+^ sensing in practical applications.

**Table 3:** Comparison of the previously reported work on carbon-based QDs with the present work for sensing of Fe^3+^ ions.

Material	Synthesis method	Linear range (µM)	LOD (µM)	Refs.
GQDs	Electrochemical exfoliation	0–80	7.22	^4^
CQDs	Thermal pyrolysis	0–300	13.68	^97^
CQDs	Hydrothermal	0–20	3.7	^98^
Carbon dots	Hydrothermal	0–500	7.4	^99^
N doped carbon dots	Hydrothermal	0–50	10.98	^10^
Carbon dots	Sonication	12.5–100	9.97	^100^
Carbon dots	Hydrothermal	0–500	28	^101^
Carbon dots	Thermal analysis	25–300	19	^10^
GQDs	Microwave treatment	0–50	2.5	Present work

## Conclusions

GQDs have been successfully synthesised through microwave treatment of biomass waste derived short range ordered carbon. The sizes of as-prepared GQDs were in the range of 5 to 20 nm and exhibited excitation independent PL emission. This phenomenon is attributed to the single-emission fluorescence centre of these GQDs. These GQD were further modified by a simple hydrothermal treatment to reduce the particle size and enhance the optical properties. The average size of modified GQDs-500-M was 1.6 ± 0.55 nm, with a very narrow size distribution. The decreased size resulted in the increase of the bandgap and PL emission in the high energy region. Moreover, a high quantum yield of 0.23 was noted for GQDs-500-M. Owing to their fascinating optical properties, GQDs-500-M were applied as PL sensor to detect Fe^3+^ ions. These GQDs were used a fluorescence probe to selectively detect Fe^3+^ ions with high sensitivity down to 2.5 ± 0.3 µM. Taken together, highly luminescent, cost-effective, highly soluble and sensitive sensing abilities (even at low concentrations) makes as prepared GQDs an ideal candidate for potential applications in sensing other metal ions and biomolecules.

## Methods

### Materials

The biomass waste, namely spent tea (PG tips), was collected from a local shop. Nitric acid (HNO_3_, > 67%) and sodium hydroxide (NaOH) were obtained from Fisher Scientific and used without modification. The salts AgNO_3_, AlCl_3_, CaCl_2_, CoCl_2_, CrCl_3_, CuCl_2_, MgCl_2_, MnCl_2_, MoCl_2_, FeCl_3_, SrCl_2_, ZnCl_2_, NaCl, LiCl and FeCl_2_ were supplied by Sigma Aldrich. The dialysis bags of 1kD MWCO (Spectrum Labs) were used for purification of GQDs. Mica discs (product code: AGF7013) and TEM grids (product code: AGS147-4H) were obtained from Agar Scientific. Deionised water was used for the preparation of all solutions.

### Characterisation

The structure and morphology of the samples were characterised by a wide range of analytical techniques. The size and microstructure of samples were studied by transmission electron microscope (FEI Titan Themis 300: X-FEG 300 kV S/TEM). A software ‘ImageJ (1.46r)’ was used to measure the particle size from TEM images. X-ray photoelectron spectroscopy (XPS) was used to analyse the surface elemental composition on a Thermo Scientific NEXSA spectrometer. Samples were analysed using a micro focused monochromatic Al X-ray source (72 W) over an area of approximately 400 µm. Data were recorded at pass energies of 200 eV for survey scans and 50 eV for high resolution scan with 1 eV and 0.1 eV step sizes, respectively. Raman spectra were obtained through a Renishaw plus Raman spectrometer using 532 nm laser excitation. Fourier transform infrared (FTIR) spectra were recorded on a Bio-Rad FTIR spectrometer (Cary 630 FTIR, Agilent Technologies) using Diamond ATR.KBr tip. The pH of the samples was monitored with the help of pH meter (Mettler Toledo, FiveEasy pH meter). The absorption and fluorescence spectra were recorded on Shimadzu UV-1800 spectrophotometer and Shimadzu RF-6000 spectrofluorophotometer, respectively.

### GQDs synthesis

GQDs were synthesised by microwave assisted thermochemical cutting of short range ordered carbon derived from pyrolysis of spent tea. Spent tea was washed, oven dried at 80 °C for 12 h and then ground to get the fine powder (< 90 µm). The obtained powder was then pyrolysed at 500 °C in inert atmosphere with a heating rate of 10 °C min^−1^ in VCTF4 furnace (Vecstar Ltd.). The pyrolysis reaction was performed for 3 h and carbon rich biochar was obtained. The biochar precursor was washed with DI water and then boiled in 0.1 M HCl to remove the impurities and reduce the ash content. The sample was again washed with DI water followed by drying in oven at 60 °C to get the purified product. An XPS analysis was performed to confirm purity of the precursor. The results indicated that the raw precursor was mainly composed of C, O and N, with traces of Ca (Fig. S2, Supplementary Information).

Next, about 20 mg of the as prepared carbon rich sample was added into the reactor containing 10 ml of DI water. About 2 ml of as received HNO_3_ was added to the reactor to create acidic conditions for oxidative cutting of carbon domains. Then the reactor was treated at a microwave power of 100–900 W for 15–180 min under reflux. Upon the completion of preadjusted duration, the obtained brown dispersion was diluted with 100 ml of DI water and filtered through 0.1 um polyvinylidene difluoride (PVDF) filtration membranes to separate the larger unreacted particles. The obtained pale-yellow filtrate contained the GQDs and hence showed bright luminescence. The synthesised GQDs required purification due to the acidic nature of the solution. The GQDs dispersion was neutralised with NaOH solution until pH 7 and then dialysed for 1 day to get the pure GQDs solution. Dry GQDs were obtained by freeze drying and a yield of up to ~ 84.5 wt.% was recorded. The synthesised GQDs were denoted as GQDs-X, where X indicates the microwave power. For example, GQDs-500 denotes the product prepared at 500 W power.

### Modification of GQDs

About 50 ml of GQDs solution obtained before purification was added to a Teflon lined autoclave for hydrothermal treatment. After hydrothermal treatment at 200 °C for 8 h, the dispersion was filtered through 0.1 µm PVDF filtration membrane followed by purification via dialysis for 1 day. The excess water was removed using rotary evaporator. The obtained concentrated solution exhibit bright luminescence and were named as GQDs-X-M, where X indicates the microwave power and M stands for modification.

### Sensing of Fe^+3^

The sensing of Fe^3+^ was performed at 340 nm of excitation using a normal quartz cuvette. The fluorescence spectra were measured using the mixture of GQDs-500-M and metal ions. In order to evaluate the selectivity of GQDs towards Fe^3+^ sensing, following fifteen different metal ions were selected: Ag^1+^, Al^3+^, Ca^2+^, Co^2+^, Cr^3+^, Cu^2+^, Mg^2+^, Mn^2+^, Mo^2+^, Fe^3+^, Sr^2+^, Zn^2+^, Na^1+^, Li^1+^ and Fe^2+^. The concentration of GQDs-500-M was kept constant at 50 μg mL^−1^ and 50 µM of each metal ion was added. After mixing for 2 min, the fluorescence spectra were measured. Quantitative measurement of Fe^3+^ was carried out by preparing a series of FeCl_3_ and GQDs-500-M solutions, in which the concentration of GQDs was kept constant but the concentration of Fe^3+^ varied from 0 to 50 µM. The fluorescence intensities of these solutions were recorded using fluorescence spectrophotometer.

## Supplementary information

Supplementary information.

## Data Availability

The data that supports the findings of this study are available from the corresponding author upon reasonable request.
