# Supplementary material for: High yield synthesis of graphene quantum dots from biomass waste as a highly selective probe for Fe3+ sensing
Source: Sci Rep. 2020 Dec 4;10:21262. doi: 10.1038/s41598-020-78070-2 (PMC7718218; doi:10.1038/s41598-020-78070-2)
Supplement: Supplementary file 1 — Supplementary information. [file 41598_2020_78070_MOESM1_ESM.pdf]

Supplementary Information

High yield synthesis of graphene quantum dots from biomass waste as a highly selective probe for Fe^{3+} sensing

Aumber Abbas[1], Tanveer A Tabish[2], Steve J Bull[1], Tuti Mariana Lim*[3] & Anh N Phan*[1]

[1] School of Engineering, Newcastle University, Newcastle upon Tyne, NE1 7RU, United Kingdom.

[2] UCL Cancer Institute, University College London, London, WC1E 6DD, United Kingdom.

[3] School of Civil and Environmental Engineering, Nanyang Technological University, 639798, Singapore.

* Corresponding authors: Tuti Mariana Lim and Anh N Phan

Email addresses: TMLim@ntu.edu.sg (T. M. Lim), anh.phan@ncl.ac.uk (A. N. Phan)

Bandgap calculation

The optical bandgap was calculated based on the UV-vis absorbance spectra using Tauc equation[1] as follows:

$$(\alpha h\upsilon)^{1/\gamma} = A(h\upsilon - E_g)$$

where, α is optical absorption coefficient, h is Planck's constant, υ is frequency of light, A is a constant and E_g is optical bandgap. The γ factor depends on the nature of material and is equal to 1/2 for direct bandgap materials and 2 for indirect bandgap materials[2]. The optical bandgap was determined from the curve of $(\alpha h\upsilon)^{1/\gamma}$ versus photon energy $h\upsilon$ converted from the UV-vis spectra. The linear segment of the curve was extrapolated to x-axis and interception point gave an estimate of the bandgap. A good linear fit was obtained while using γ = ½ and no good fit was obtained when γ = 2 was used, as shown in Fig. S1. These results indicate a direct bandgap of GQDs studied in this work.

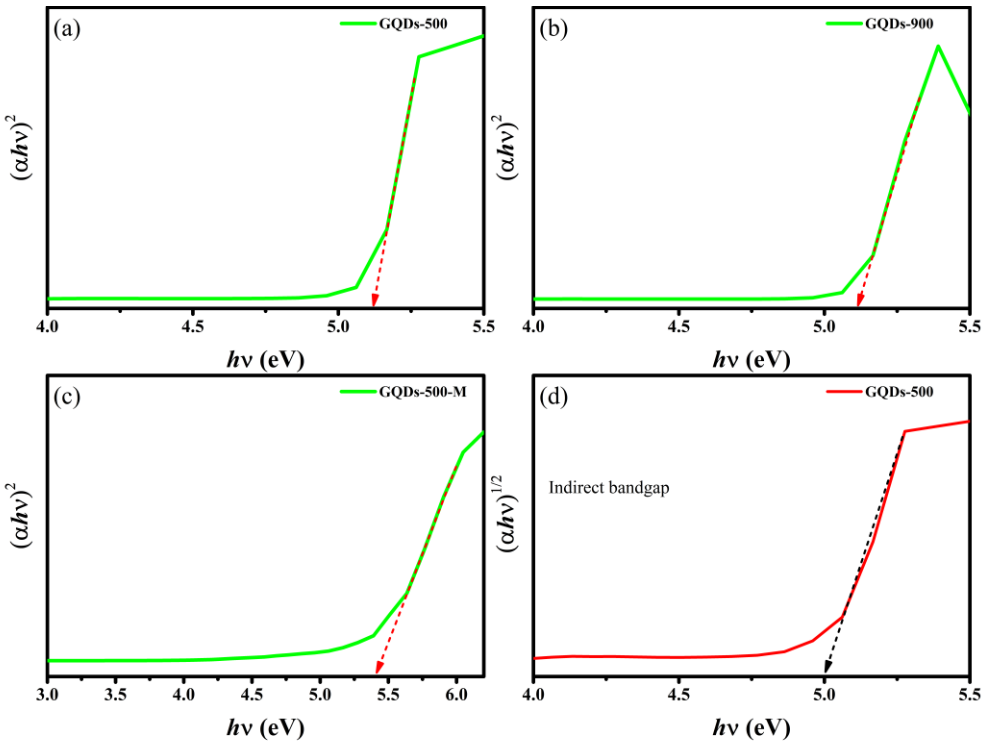

Fig. S1. Optical bandgap curves derived from Tauc equation. Linear part of the plot is extrapolated to x-axis. Direct bandgap calculation of (a) GQDs-500, (b) GQDs-900, (c) GQDs-500-M and (d) indirect bandgap determination for GQDs-500. Drawings were plotted using OriginPro 2018b (https://www.originlab.com).

The direct bandgap values of GQDs-500, GQDs-900 and GQDs-500-M were calculated to be ~5.12, ~5.12 and ~5.4 eV, respectively.

Precursor purification

The biochar precursor obtained after pyrolysis was washed with hot DI water (90 °C) and then boiled in 0.1 M HCl to remove the impurities and reduce the ash content. The sample was again washed with DI water until almost neutral pH was obtained in filtrate. Finally, the washed product was dried in oven at 60 °C overnight and purified product was obtained. An XPS analysis was performed to confirm purity of the precursor (Fig. S2). The XPS survey spectrum indicates four major peaks at ~284.4, ~400.4, ~532.4 and ~347.4 eV corresponding to C1s, N1s, O1s and Ca 2p. Another small peaks at ~438 eV is related to Ca 2s. These results indicate that the raw precursor is mainly composed of C, O and N, with limited content of Ca from the feedstock. Ash content in the biomass derived biochar precursor is the origin of calcium and it is present as $CaCO_3$-like species[3].

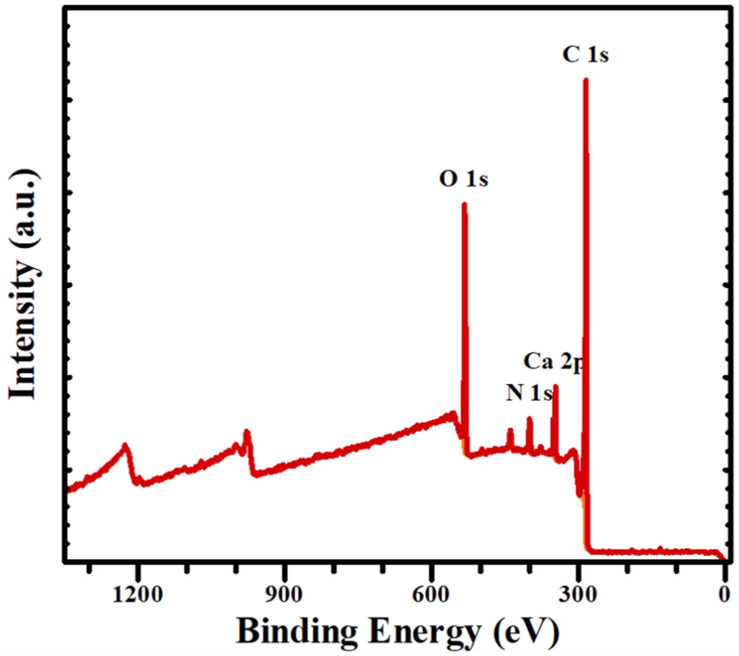

Fig. S2. XPS survey spectrum of purified biochar precursor indicating four major peaks corresponding to C1s, O1s, N1s and Ca2p. Figure was drawn using OriginPro 2018b (https://www.originlab.com).

References

1 Tauc, J., Grigorovici, R. & Vancu, A. Optical properties and electronic structure of amorphous germanium. *Phys. Status Solidi B* **15**, 627-637 (1966).

2 He, S., Turnbull, M. J., Nie, Y., Sun, X. & Ding, Z. Band structures of blue luminescent nitrogen-doped graphene quantum dots by synchrotron-based XPS. *Surf. Sci.* **676**, 51-55 (2018).

3 Dai, L. *et al.* Calcium-rich biochar from the pyrolysis of crab shell for phosphorus removal. *J. Environ. Manage.* **198**, 70-74 (2017).
